# Morphometric examination of trigeminal nerve and its adjacent structures in patients with trigeminal neuralgia: a case-control study

**DOI:** 10.55730/1300-0144.5503

**Published:** 2022-07-28

**Authors:** Yunus Emre KUNDAKCI, Nadire ÜNVER DOĞAN, İnci KARA, Mehmet ÖZTÜRK, Zeliha FAZLIOĞULLARI, Ahmet Kağan KARABULUT

**Affiliations:** 1 Department of Physiotherapy and Rehabilitation, Faculty of Health Sciences, Afyonkarahisar Health Sciences University, Afyonkarahisar, Turkey; 2 Department of Anatomy, Faculty of Medicine, Selçuk University, Konya, Turkey; 3 Department of Algology, Faculty of Medicine, Selçuk University, Konya, Turkey; 4 Department of Radiology, Faculty of Medicine, Selçuk University, Konya, Turkey

**Keywords:** Trigeminal neuralgia, facial pain, brain stem, anatomy, morphometry

## Abstract

**Background/aim:**

Morphological differences that can lead the trigeminal nerve to neurovascular conflict and a new solitary pontine lesion are associated with the pathogenesis of trigeminal neuralgia (TN). In this case-control study, we aimed to contribute to the current discussions about the pathogenesis of TN by investigating the anatomical structures that may have an effect on the morphometric parameters of the trigeminal nerve.

**Materials and methods:**

This study included 25 patients with TN followed up for pain in the Department of Algology, Faculty of Medicine, and 25 age- and gender-matched controls. We performed morphometric measurements including the length and volume of the trigeminal nerve, cerebellopontine cistern, pons, and posterior fossa in the MRIs of these individuals. Comparative analyses were performed for the mean of the affected and unaffected sides of the TN patients and the right, left, and both sides of the control group.

**Results:**

In patients with TN, on the affected side, length and volume of the trigeminal nerve and cerebellopontine cistern volume were found smaller than controls (p < 0.05). Pons volume was higher in patients with TN compared to controls (p < 0.05). The length of the affected nerve was significantly related to prepontine cistern length and cerebellopontine cistern volume (p < 0.05).

**Conclusion:**

The cerebellopontine cistern volume has a significant impact on the morphometric characteristics of the trigeminal nerve. Especially, whether the increase in the volume of pons causes a decrease in the volume of cerebellopontine cistern should be clarified with further research.

## 1. Introduction

Trigeminal neuralgia (TN), also known as tic douloureux, is an electric shock-like or stabbing chronic neuropathic pain disorder that occurs in a region of the face [1]. The ophthalmic (V1), maxillary (V2), and mandibular (V3) branches of the trigeminal nerve arise from the skull base, and pain most commonly affects the distribution of the V2 and V3 segments [1,2]. Depending on the severity of the pain, the quality of life of patients with TN decreases, and even daily activities such as talking, washing face, and chewing are sometimes restricted [1,3]. Painful periods or measures taken for a pain-free life affect not only the patient but also the family. Because of these clinical features, TN continues to challenge patients, their families/caregivers, and health care providers [4].

Various theories have been presented in the underlying pathophysiology of TN. Neurovascular conflict (NVC) between the trigeminal nerve and adjacent blood vessels is believed to be the most common cause of TN [5]. MRI and diffusion tensor imaging studies have highlighted an NVC with morphometric alterations, dysregulation of voltage-gated sodium channel expression in the membrane, and dysmyelination or demyelination in the microstructure of the trigeminal nerve [6–8]. In addition, advances in the classification of TN have indicated that classical TN contains a combination of NVC and morphological changes in the trigeminal nerve, whereas idiopathic TN has no morphological changes in the trigeminal root with or without NVC [9–11].

Over the past few decades, there has been increasing evidence that NVC does not explain disease conditions in all TN patients, and those healthy individuals may also have NVC [5, 12, 13]. Previous studies have indicated that adjacent neuroanatomical variations of the trigeminal nerve [14,15], concomitant persistent pain [16], and genetic factors [17,18] may play an important role in the pathophysiology of TN without NVC. On the other hand, the etiology of a smaller trigeminal nerve on the affected side in TN, previously defined as nerve atrophy, remains unclear [15,19,20]. This has led to careful consideration of other factors that predispose or contribute to TN, including anatomical differences in the brain.

Volumetric neuroimaging studies have revealed smaller posterior fossa and cerebellopontine cistern volumes and increased posterior fossa crowding in TN [14, 15, 21]. Most of these studies have examined nerve atrophy, and few have focused on the NVC status [15]. Furthermore, the relationship between nerve atrophy assessed by volumetric measurements and neuroanatomical structures adjacent to the nerve has not been previously addressed in patients and controls without NVC. Neuroanatomical abnormalities in the brain may contribute to the development of NVC and lead to morphometric changes in the nerve. These conditions especially need to be understood independently of the NVC status.

The aim of this study was to provide further information regarding the etiology of this disease by considering the presence of trigeminal nerve atrophy in clinical conditions other than severe NVC and the relationships between brain structures that may cause morphometric differences in the nerve. Therefore, this study focused on revealing the morphometric differences of the trigeminal nerve in the absence of severe NVC, clarifying nerve atrophy, and the relationship of these conditions with adjacent brain anatomical structures.

## 2. Materials and methods

### 2.1. Study design

We performed a case-control study based on MRI morphometric measurements of anatomical structures of the brain, such as the trigeminal nerve, cerebellopontine cistern, pons, and posterior fossa. As in a previous study [15], groups were matched for age and gender to minimize the effect of age-related changes in brain volume. In addition, the presence of severe NVC was not reported by the neuroradiologist for all individuals in the patient and control groups. This study was conducted in accordance with the principles published in the Helsinki Declaration of Human Rights. The study protocol was approved by the Clinical Research Ethics Committee of the university (approval number: 2019/243).

### 2.2. Study population

**Figure 1:**
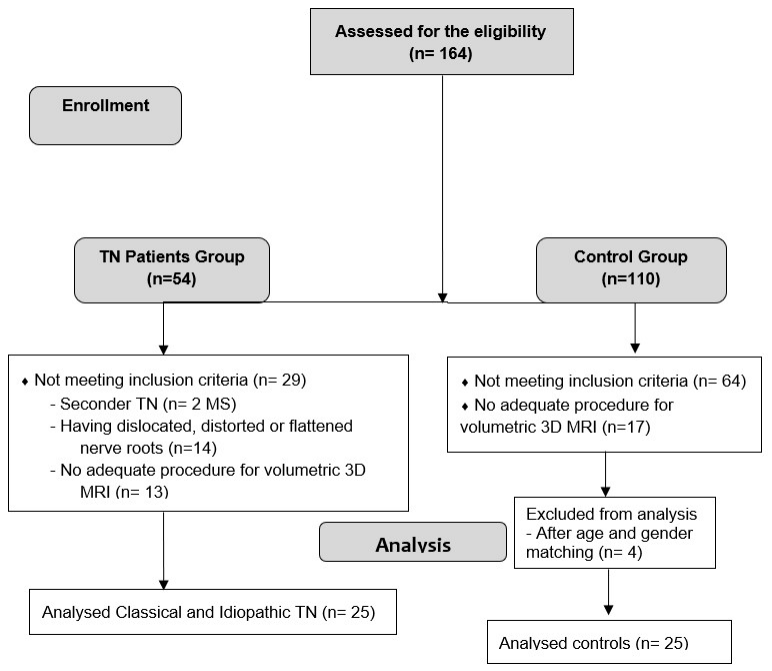
Flow diagram of the study. 3D MRI; three-dimensional magnetic resonance imaging, MS; multiple sclerosis.

In this study, the TN group consisted of 54 consecutive patients who applied to the Faculty of Medicine, Department of Algology between 2015–2019. The control group was selected from 110 individuals with brainstem radiological images in the radiology department archive and who met the study criteria. The inclusion criteria were clinically established TN, including idiopathic and classic TN, except for secondary TN [9–11]. In the International Classification of Headache Disorders-3 classification [11], since the severity of NVC affects the diagnostic criteria, simple contact between the trigeminal nerve and a vessel was defined as NVC. A contact involving dislocated, distorted or flattened nerve roots on MRI was determined as severe NVC. In these classic TN patients with severe NVC, the primary factor causing the morphological change was considered to be vascular compression and was not included in the study. Other exclusion criteria were bilateral symptoms, diagnosis of orofacial pain other than TN under the age of 18, previous microvascular decompression surgery for TN, neurological and/or psychological symptoms, a history of surgery in the area to be measured, or a secondary deficit that may cause pathoanatomical changes. We also examined the MRI of 110 controls based on similar exclusion criteria as the patient group and matched them with patients for age and gender. Finally, the case-control study groups consisted of 25 patients (25 to 87 years) and 25 controls (19 to 78 years) (Figure 1).

### 2.3. Brain MRIs

All data were obtained on a 1.5T MRI scanner (Siemens Magnetom Aera; Siemens AG Healthcare Sector, Erlangen, Germany) using a standard head coil. Parameters for T1-W axial images were as follows: FOV: 230 mm, matrix: 256 × 256, slice thickness: 5 mm, interslice gap: 1 mm, NEX: 2–3, TR/TE: 562/14 ms. The CISS sequence parameters were TR (repetition time), 5.42 ms; TE (echo time), 2.42 ms; flip angle, 77°; bandwidth, 399 Hz/Px; slice thickness, 0.70 mm, with an interslice gap of 20% and with 64 slices in a single slab; matrix size, 256 × 256. A TE of 2.42 ms was chosen to limit signal loss from magnetic susceptibility effects, and a flip angle of 77° was used to reduce T1 weighting. The acquisition time was 3.30 min with the use of two averages. TOF MRA was obtained using 3D gradient echo sequences (TE 7.15 ms, TR 24 ms, flip-angle 25°, multiple overlapping thin slab acquisition, in-plane pixel spacing 0.625 mm, slice spacing 0.8 mm, slab thickness 28.8 mm; Cases 2 and 3: TE 3.2 ms, TR 25 ms, flip-angle 20°, with variable flip-angle excitation and magnetization transfer contrast, in-plane pixel spacing 0.352 mm, slice spacing 0.6 mm). No gadolinium contrast was administered.

### 2.4. Morphometric analyses

All images were transferred to a postprocessing Workstation (SyngoVia (VB30A), Siemens Healthineers, Erlangen, Germany) to calculate volume and length measurements. Multiplanar reconstruction was utilized on the workstation to evaluate neuroanatomical structures. Images were reconstructed in the sagittal, coronal, and axial planes. The reformatted images were magnified to facilitate the measurement of the length and volume of the target. Morphometric length and volume parameters were determined and measured as previously described [15, 22, 23]. The length measurements were calculated by manually drawing on axial images with a section thickness of 0.5 mm, and the volume measurements were calculated automatically with the 3D reconstruction software. All measurements were performed by an anatomist blind to the affected side of the TN and clinical data. Additionally, an experienced radiologist confirmed the accuracy of the measurements and the absence of severe NVC in the study population.

**Figure 2:**
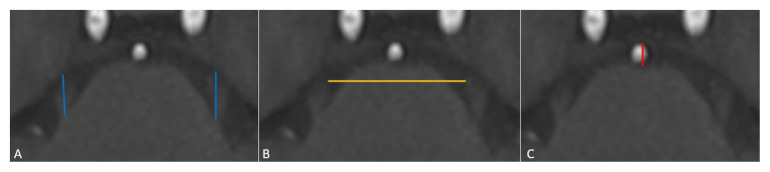
The figure shows the limits used in the evaluation of morphometric length measurements (cm) on T1-weighted MRI scans in a 39-year-old female patient with TN. (**A**); trigeminal nerve length (blue draws), (**B**); nerve-to-nerve distance (yellow draw), (**C**); anterior-posterior prepontine cistern length (red draw).

The length of the cisternal segment of the trigeminal nerve was defined as the length from the point where the nerve emerges from pons to Meckel’s cave, and a slice was selected showing the greatest length of both trigeminal nerves passing through the cistern (Figure 2A). The distance between the right and left trigeminal nerves (nerve-to-nerve distance) was determined by drawing a straight line between the most medial limits of each nerve (Figure 2B). The anteroposterior length of the prepontine cistern was determined by measuring a straight line in the midline between the most posterior limit of the clivus and the most anterior limit of the pons (Figure 2C).

**Figure 3:**
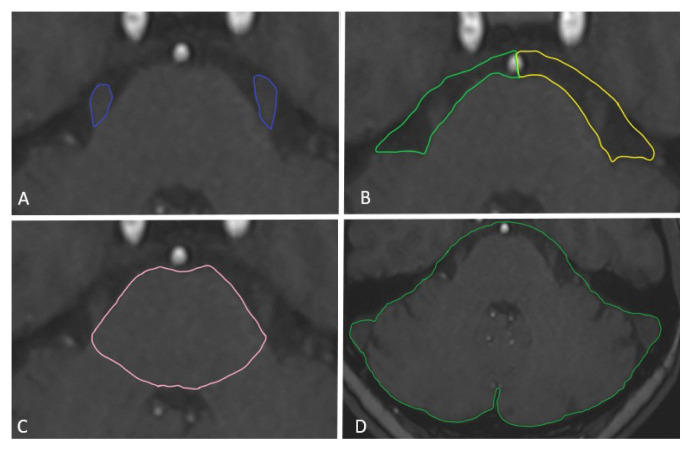
The figure shows the exemplary anatomical limit of the brain structures evaluated for volume measurements (cm^3^) on T1-weighted MRI scans in a 39-year-old female patient with TN. All volume measurements were automatically calculated on images transferred to a postprocessing Workstation (syngo.via version VB30A, Siemens Healthcare). (**A**); trigeminal nerve volume (blue area), (**B**); cerebellopontine cistern volume (yellow and green areas). (**C**); pons volume (pink area), (**D**); posterior fossa volume (green area).

The superior and inferior limits of the slices containing the trigeminal nerve were determined for volumetric measurements, except for the posterior fossa volume. In addition, the slice containing the clearest image of the trigeminal nerve was determined as the middle limit. The volumes of the trigeminal nerve, pons, and cerebellopontine cistern covered the area limited by the determined superior, middle and inferior slices. The volumes of the cerebellopontine cistern and trigeminal nerve were measured bilaterally. The volume of the trigeminal nerve was measured by drawing in a limited area in the selected superior, middle, and inferior slices, and then transferred to the workstation for automatic volume calculation (Figure 3A). The cerebellopontine cistern was defined as the area between the anterior surface of the pons and cerebellum and the posterior surface of the petrous bone. The cerebellar flocculus was defined as the posterior limit and the midline as the anterior limit. The median line was determined by drawing a line between the anterior median fissure and the median sulcus of the floor of the fourth ventricle. The volume of the cerebellopontine cistern was measured by limiting it to the determined superior, middle, and inferior slices, and then transferred to the workstation for automatic volume calculation (Figure 3B).

In pons volume measurements, the anterior margin was the anterior surface of the pons, the posterior margin was the posterior surface of the pons as it abuts the fourth ventricle, and the lateral limits were the most lateral surfaces of the pons, not including the middle cerebellar peduncles. The volume of the pons was measured by limiting it to the determined superior, middle, and inferior slices, and then transferred to the workstation for automatic volume calculation (Figure 3C).

In the posterior fossa volume measurements, the most inferior limit of the posterior fossa was the most inferior slice containing the cerebellar tonsils. The superior limit was the most superior slice containing a trigeminal nerve. All other limits were the bony margins of the posterior fossa. Similarly, measurements for posterior fossa volume were then transferred to the workstation for automatic calculation (Figure 3D). In addition, in order to examine the morphometric differences in the trigeminal nerve depending on the duration of the symptoms, the difference of the values obtained from the measurements between the affected side and the unaffected side of the patients with TN was calculated.

### 2.5. Statistical analysis

The sample size was calculated using the G*Power 3.1.3 program (Statistical power (1−β) = 0.95, significance level α = 0.05, effect size f = 0.75, number of groups = 2). In Wilcoxon T analysis, a calculation was made on 22 samples for each group and 25 samples were selected for each group, taking into account the elimination rate.

Continuous variables were tested for a normal distribution with the Shapiro-Wilk test. A paired samples t-test or independent samples t-test was used to compare the two groups. Variables that had a nonhomogenous distribution were compared with a Wilcoxon signed-rank test (for dependent variables) and Mann–Whitney-U test (for independent variables). In within-group comparisons, affected and unaffected sides (symptomatic and asymptomatic sides) of TN patients and right and left sides of controls were included. Comparative analyses were also performed between the patient and control groups (affected/unaffected/control). Pearson correlation test was used to examine the correlations between morphometric parameters on the affected side of TN patients and various factors (symptom duration and age). In addition, the same test was used to correlate the difference in morphometric measures of TN patients with the duration of symptoms. All analyses were performed with SPSS 25.0 with the significance level set to p < 0.05.

## 3. Results

### 3.1. Demographic data

**Figure 4:**
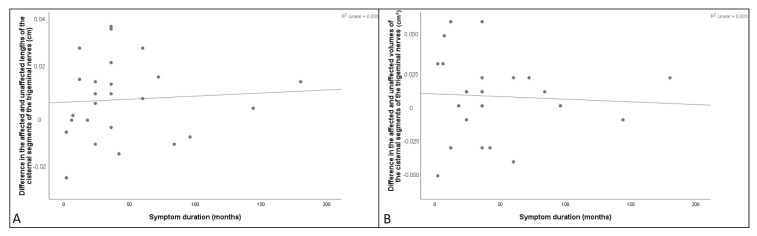
The correlation scatter plots. **(A)** Correlation of the difference in the affected and unaffected lengths of the cisternal segments of the trigeminal nerves with symptom duration (r = 0.069, p = 0.744). **(B)** Correlation of the difference in the affected and unaffected volumes of the cisternal segments of the trigeminal nerves with symptom duration (r = −0.058, p = 0.784).

Twenty-five patients with TN (21 females and 4 males) and 25 controls (21 females and 4 males) with MRIs were included in this study. There was no statistical difference in the mean age of patients with TN and controls (p > 0.05). Twelve patients had pain on the right side and 13 patients had pain on the left side. The V3 division (48%) was the part most affected. Seventeen (68%) patients were being followed up with only medical therapy. The duration of symptoms ranged from 2 to 180 months (mean 44.4 months, 95% CI 26.5–62.3) (Table 1). In addition, the distribution of symptom durations is presented in Figure 4.

### 3.2. Length and volume of the cisternal segment of the trigeminal nerve

**Figure 5:**
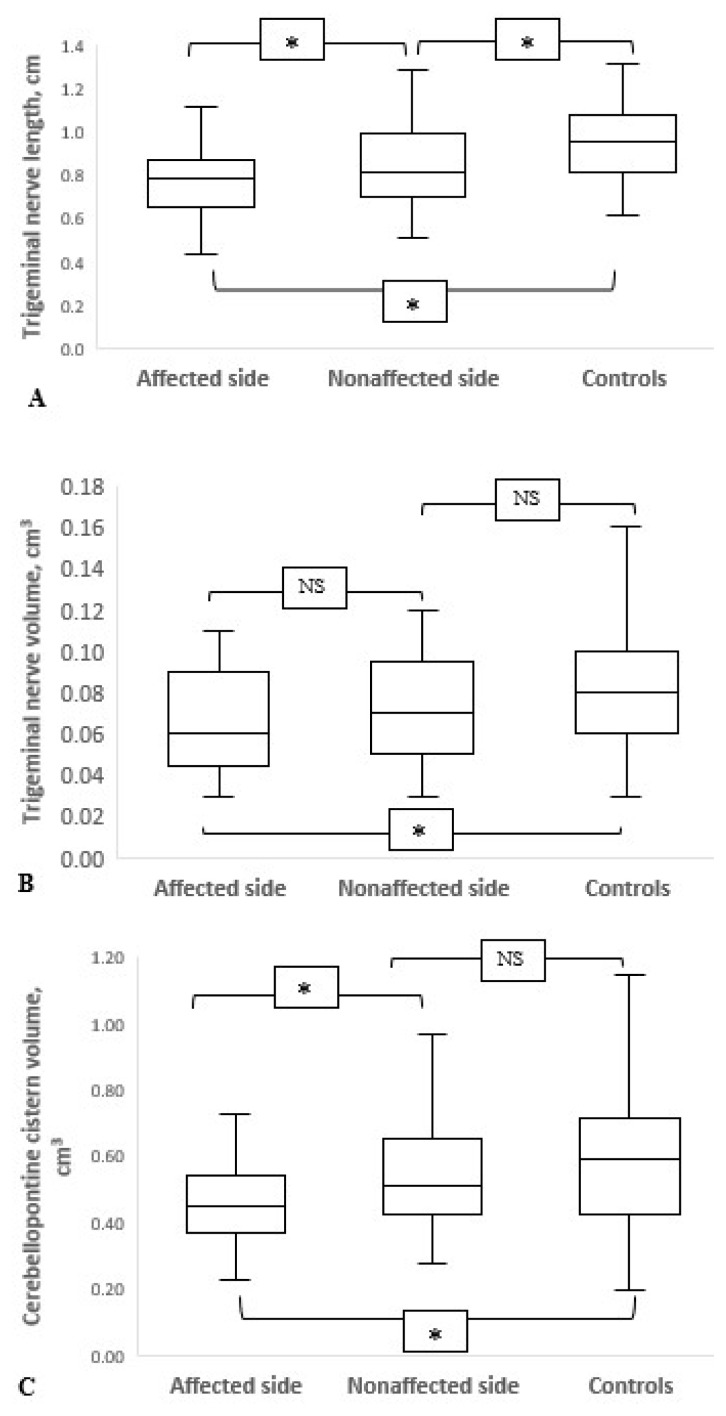
Comparison of the affected (n = 25) and unaffected (unaffected) sides (n = 25) of TN patients with the mean values of the right and left (n = 50) total of the control group. (**A**) The length of cisternal segment of the trigeminal nerve (cm), (**B**) The volume of cisternal segment of the trigeminal nerve (cm^3^), (**C**) The volume of the cerebellopontine cistern (cm^3^). * Statistical significance at the p < 0.05 level. NS, not significant.

In patients with TN, the trigeminal nerve length was significantly decreased on the affected side (p = 0.028). There was no difference between the right and left sides in the controls (p = 0.815) (Table 2). In addition, controls had longer nerves on either side (affected and unaffected sides) than TN patients (p < 0.001; p = 0.014, respectively) (Figure 5A).

In within-group comparisons, neither patients nor controls showed statistically significant differences in trigeminal nerve volumes on either side (p = 0.252; p = 0.107, respectively) (Table 2). However, it was found that the trigeminal nerve volume on the affected side of the patients was statistically significantly decreased compared to the controls (p = 0.04) (Figure 5B).

### 3.3. Cerebellopontine cistern, pons and posterior fossa volumes

The cerebellopontine cistern volume was significantly higher on the unaffected side than on the affected side in patients with TN (p = 0.005) (Table 2). However, there was no statistically significant difference between right and left cerebellopontine cistern volumes in controls (p = 0.915) (Table 2). In addition, only the cerebellopontine cistern volume of the affected side was found to be significantly lower in patients compared to controls (p = 0.004) (Figure 5C). The pons volume was significantly higher in TN patients than in controls (p = 0.017). Finally, there was no difference in posterior fossa volume between the TN and control groups (Table 3).

### 3.4. Correlation of duration of symptoms, age, and morphometric parameters in patients with TN

It was observed that the mean cisternal nerve length of the affected side was positively correlated with the AP length of the prepontine cistern (r = 0.485, p = 0.014) and the volume of the cerebellopontine cistern of the affected side (r = 0.477, p = 0.016) (Table 4). In addition, a significant positive correlation was found between the volume of the cerebellopontine cistern of the affected side and the AP length of the prepontine cistern (r = 0.510, p = 0.009) (Table 4). There was no significant correlation between duration of symptoms, age, and morphometric parameters on the affected side in patients with TN (p > 0.05) (Table 4). In Figure 4, the correlation between symptom duration and values of length and volume difference is presented in a scatterplot. No significant correlation was observed between bilateral length and volume difference values and symptom duration in TN patients (p > 0.05).

## 4. Discussion

This study suggests that changes in trigeminal nerve volume in TN patients are associated with different morphometric features of adjacent brain structures. Examining the morphometric brain structures of individuals without NVC using volumetric measurements, our current study demonstrates significant differences in the cerebellopontine cistern and pons volumes in TN compared to controls. In the brain of a TN patient without severe NVC, the length of the trigeminal nerve on the affected side and the cerebellopontine cistern volume on the affected side are reduced. Compared with age, gender, and NVC status matched controls, we observe smaller trigeminal nerve length, decreased trigeminal nerve volume on the affected side, decreased cerebellopontine cistern volume on the affected side, and increased pons volume.

There have been increasing reports examining the role of the neuroanatomical structures contributing to TN pathophysiology [14,15,19–21]. In some studies, posterior fossa volume and cerebellopontine cistern area were not associated with TN [24–26]. In contrast to these reports, there were studies that primarily associated a smaller posterior fossa [14,15,21] and a smaller cerebellopontine cistern [15,21,27–30] with the disease. On the other hand, differences in pons structure caused by solitary pontine lesions and texture abnormalities of the brainstem at the level of the pons were emphasized in several recent studies [31,32]. Interestingly, no morphometric difference in the pons with TN has been previously reported. This study demonstrated that the cerebellopontine cistern on the affected side may shrink and the pons may enlarge in TN patients without NVC. In contrast, one study reported that pons volume was similar in patients and controls [15]. At this point, it may be useful to evaluate the morphometric findings of the structures adjacent to the nerve together. There may be an anatomical structural relationship that supports our findings: A larger pons may narrow the cerebellopontine cistern in patients with TN without NVC. This can also cause a decrease in the volume and length of the trigeminal nerve. A similar association with crowded and smaller cerebellopontine space in the posterior cranial fossa has recently been demonstrated [21]. These results go beyond previous reports, showing that the morphometric structure of the pons in patients with trigeminal neuralgia should be examined more comprehensively. Considering the cisternal segment of the trigeminal nerve and the anatomical features of the structures adjacent to it together, all of these may be significant observations that indeed may predispose patients to the development of TN with appropriate vascular conflict.

One study reported that trigeminal nerve root atrophy was not associated with age or disease duration in classical and idiopathic TN [16]. In line with the previous study, we found that morphometric differences in trigeminal nerve were unrelated to symptom duration. This finding may be due to the absence of severe NVC in patients included in the current study.

A previous study reported an association between the cross-sectional area of the cerebellopontine cistern and the length of the cisternal segment of the trigeminal nerve [28]. In this study, the length of the trigeminal nerve and the volume of the cerebellopontine cistern showed a similar relationship. We attributed this to the anatomical features of the nerve and cistern. The cisternal segment of the trigeminal nerve, as is well known, arises from the anterolateral aspect of the pons, then courses through the prepontine cistern into the Meckel’s cave [2,33].

Cheng et al. reported that primary TN patients had a more crowded posterior fossa than controls [14]. Hardaway et al. reported no significant difference in posterior fossa volume between TN patients and controls but found greater posterior fossa volume in TN patients with NVC than in those without NVC. In the same study, they reported a smaller posterior fossa volume in men with TN without NVC [15]. The findings in previous studies [14,15,21] that the morphometric structure of the posterior fossa increases the risk of NVC were not demonstrated in this current study. The reason for these differences can be attributed to the presence or absence of NVC between studies.

Anatomical alterations in the trigeminal root, such as distortion or atrophy, are associated with the pathophysiology of TN rather than simple contact between nerve and vascular structure [1]. Morphologic changes are rarely seen on the asymptomatic side [6,34]. The current study has demonstrated that TN without severe NVC may not always involve atrophic changes. The results of the present study were consistent with several studies [6,35,36]. Studies have reported that atrophic changes in the trigeminal nerve are usually seen with severe NVC [6] and that most TN patients with NVC do not have nerve atrophy [36]. In addition to all these, nerve atrophy has been frequently shown in many studies in the literature, primarily involving trigeminal nerve morphometry. Except for a few studies in the literature [24,25,35], differences in the length and volume of the trigeminal nerve on the affected side have been reported in TN patients [7,19,22,26–30,37] (Table 5). Volumetric 3D analyses provide a more effective assessment of nerve atrophy compared to length and diameter measurements [22,23]. Therefore, we considered the major structural change in the trigeminal nerve on the affected side was a morphological volume decrease. No trigeminal nerve atrophy was observed in the patients in this present study, using similar methods with morphometric studies and comparing the mean between similarly affected and unaffected sides. However, our results show that the volume of the trigeminal nerve is significantly lower on the affected side compared to controls. Different findings in the literature may vary depending on the presence of NVC in patients, as well as depending on the patient and control sample.

The area and volume of the trigeminal nerve are the clinicians’ anatomic keys in the diagnosis and treatment of patients with TN [10]. In this study, morphological changes in the cistern segment of the trigeminal nerve and its adjacent anatomical structures were discussed in detail in light of the literature. While interpreting the results of the study, we demonstrated that the proximity between anatomical structures may be effective in trigeminal nerve measurements. To the best of our knowledge, this is the first study to evaluate the morphometric differences in the trigeminal nerve and brainstem structures in patients without severe NVC and in controls and to methodologically investigate the relationship between anatomical structures using 3D methods. In this context, the findings of the study can contribute to the literature by considering the current limitations.

The current study has some limitations. The number of subjects in our study was relatively small compared to other studies in the literature that included patients without NVC. Although age and gender matches were achieved between the patient and control groups, the inability to evaluate the effect of body size on radiological measurements due to the retrospective design and incomplete hospital records can be seen as a limitation. Therefore, it is difficult to generalize the results. Better-designed studies should be conducted to confirm the current findings. There are many methods to evaluate morphometric parameters in the literature. In this study, we selected a few of them. We also evaluated radiological images using T1-weighted images rather than high-resolution T2-weighted images. All these methodological differences should be taken into account when comparing existing studies with other studies.

In summary, current findings suggest that changes in the cerebellopontine cistern and pons should also be considered in the evaluation of trigeminal nerve morphometry in patients with TN. As a matter of fact, the reason for a reduced cerebellopontine cistern volume cannot be demonstrated by a potential large pons volume alone. Anatomic structural abnormalities in the pons, which is the origin of the cisternal segment of the trigeminal nerve, and the Meckel’s cave, where it terminates, should be investigated. The effect of larger pons volume and smaller cerebellopontine cistern on the morphometry of the trigeminal nerve should be evaluated comprehensively, in a larger number of patients.

## Figures and Tables

**Table 1:** Demographic data of the patients with TN and controls.

	Patients (n = 25)	Controls (n = 25)
Male (n)	4	4
Female (n)	21	21
Mean age ± SD; range (yrs)	54 ± 14.06 (25–87)	51.12 ± 12.07 (19–78)
Duration of symptoms ± SD; range (mos)	44.36 ± 43.37 (2–180)	-
**Affected (symptomatic) side and branches**	**Right (n = 12)**	**Left (n = 13)**
V1	-	-
V2	1	1
V3	5	7
V1 + V2	1	1
V2 + V3	5	3
V1 + V2 + V3	-	1
**Treatment protocols**	**Right (n = 12)**	**Left (n = 13)**
MT + RT	1	3
MT+ NB	4	-
MT	7	10

SD, standard deviation; MT, medical therapy; RT, radiofrequency thermocoagulation NB, nerve block; V, trigeminal nerve; V; 1. ophthalmic; 2. maxillary; 3. mandibular nerve.

**Table 2:** Comparisons of the affected and nonaffected sides in patients with TN and controls.

	Trigeminal nerve length (cm), Mean ± SD (Range)	Trigeminal nerve volume (cm^3^), Mean ± SD (Range)	Cerebellopontine cistern volume (cm^3^), Mean ± SD (Range)
**Patients (n = 25)**			
Affected	0.78 ± 0.19 (0.44–1.2)	0.07 ± 0.02 (0.03–0.11)	0.47 ± 0.14 (0.23–0.89)
Nonaffected	0.85 ± 0.21 (0.51–1.29)	0.07 ± 0.03 (0.03–0.12)	0.54 ± 0.16 (0.28–0.97)
p-value	0.028*	0.252	0.005*
**Controls (n = 25)**			
Mean	0.97 ± 0.18 (0.62–1.32)	0.08 ± 0.03 (0.03–0.16)	0.6 ± 0.21 (0.2–1.15)
Right	0.97 ± 0.17 (0.64–1.31)	0.08 ± 0.03 (0.03–0.16)	0.6 ± 0.21 (0.2–0.96)
Left	0.98 ± 0.2 (0.62–1.32)	0.08 ± 0.02 (0.04–0.12)	0.59 ± 0.21 (0.25–1.15)
p-value	0.815	0.107	0.915

SD, standard deviation.

* Statistical significance.

* Statistical significance at the p < 0.05 level. NS, not significant.

**Table 3:** Comparisons of length and volume morphological values in between groups.

Variables and groups	Mean ± SD (Range)	p value
Nerve-to-nerve distance (cm)		
Patients (n = 25)	3.1 ± 0.31 (2.4–3.49)	0.467
Controls (n = 25)	3.19 ± 0.21 (2.78–2.58)	
AP prepontine cistern length (cm)		
Patients (n = 25)	0.57 ± 0.17 (0.35–1.02)	0.854
Controls (n = 25)	0.57 ± 0.12 (0.39–0.84)	
Pons volume (cm^3^)		
Patients (n = 25)	2.7 ± 0.42 (1.7–3.5)	0.017*
Controls (n = 25)	2.46 ± 0.44 (1.83–3.33)	
Posterior fossa volume (cm^3^)		
Patients (n = 25)	138,12 ± 15,37 (110.9–167.11)	0.856
Controls (n = 25)	137,34 ± 14.64 (111.45–163.11)	

SD, standard deviation; AP, anterior-posterior.

* Statistical significance.

**Table 4:** Correlations of the affected side in patients with TN.

	DS	Age	TNL	NND	PCL	TNV	CPCV	PV
**Age**	0.284							
**TNL**	0.140	0.024						
**NND**	0.082	0.032	0.377					
**PCL**	−0.239	−0.343	**0.485** ^*^	−0.082				
**TNV**	−0.110	0.145	0.335	0.385	0.040			
**CPCV**	−0.223	0.289	**0.477** ^*^	0.009	**0.510** ^**^	0.357		
**PV**	0.085	0.112	0.147	0.371	−0.030	0.046	0.012	
**PFV**	−0.038	0.332	−0.182	−0.050	−0.010	−0.138	0.150	0.230

Statistical significance, p < 0.05^*^; p < 0.01^**^. Pearson correlation coefficients (r) are presented in the table.

CPCV, cerebellopontine cistern volume; DS, duration of symptoms; NND, nerve-to-nerve distance; PCL, anterior-posterior prepontine cistern length; PFV, posterior fossa volume; PV, pons volume; TNL, trigeminal nerve length; TNV, trigeminal nerve volume.

**Table 5:** Summary of anatomical measurements for volume and length of the trigeminal nerve in patients with TN and controls.

	N		Difference in mean volume			Difference in mean length		
Author	TN	C	Affected vs. unaffected	Right vs. left	Patients vs. controls	Affected vs. unaffected	Right vs. left	Patients vs. controls
Cheng et al. (2017)	60	30	↓	o	↓	o	o	o
Park et al. (2009)	26	–	↓	–	–	↓	–	–
Hořínek et al. (2009)	18	15	↓	–	O	–	–	–
Leal et al. (2014)	50	20	↓	O	↓	–	–	–
Park and Ha (2015)	30	30	o	–	O	–	–	–
Wilcox et al. (2013)	9	26	o	–	↓	–	–	–
Wang et al. (2016)	40	40	↓	–	↓	–	–	–
Leal et al. (2011)	10	6	↓	–	↓	–	–	–
Günesli and Tufan (2020)	104	98	–	–	–	↓	o	–
Pang et al. (2019)	25	25	–	–	–	o	–	o
Ha et al. (2012)	30	30	–	–	–	↓	–-	↓
Parise et al. (2013)	26	18	–	–	–	↓	–	o
Hardaway et al. (2019)	232	100	–	–	–	-	–	↓
Present study	25	25	o	o	↓	↓	o	↓

N, patient number; TN, trigeminal neuralgia; C, control; “↓”, significant decrease (on the affected side, in patients); “o”, not significant; “-“, not studied.
